# Targeting SREBP-2-Regulated Mevalonate Metabolism for Cancer Therapy

**DOI:** 10.3389/fonc.2020.01510

**Published:** 2020-08-21

**Authors:** Linyuan Xue, Hongyu Qi, He Zhang, Lu Ding, Qingxia Huang, Daqing Zhao, Boyang Jason Wu, Xiangyan Li

**Affiliations:** ^1^Research Center of Traditional Chinese Medicine, College of Traditional Chinese Medicine, Changchun University of Chinese Medicine, Changchun, China; ^2^Key Laboratory of Active Substances and Biological Mechanisms of Ginseng Efficacy, Ministry of Education, Jilin Provincial Key Laboratory of Bio-Macromolecules of Chinese Medicine, Jilin Ginseng Academy, Changchun University of Chinese Medicine, Changchun, China; ^3^College of Traditional Chinese Medicine, Changchun University of Chinese Medicine, Changchun, China; ^4^Department of Pharmaceutical Sciences, College of Pharmacy and Pharmaceutical Sciences, Washington State University, Spokane, WA, United States

**Keywords:** SREBP-2, HMG-CoA reductase, mevalonate, cholesterol, cancer therapy

## Abstract

Recently, targeting metabolic reprogramming has emerged as a potential therapeutic approach for fighting cancer. Sterol regulatory element binding protein-2 (SREBP-2), a basic helix-loop-helix leucine zipper transcription factor, mainly regulates genes involved in cholesterol biosynthesis and homeostasis. SREBP-2 binds to the sterol regulatory elements (SREs) in the promoters of its target genes and activates the transcription of mevalonate pathway genes, such as HMG-CoA reductase (HMGCR), mevalonate kinase and other key enzymes. In this review, we first summarized the structure of SREBP-2 and its activation and regulation by multiple signaling pathways. We then found that SREBP-2 and its regulated enzymes, including HMGCR, FPPS, SQS, and DHCR4 from the mevalonate pathway, participate in the progression of various cancers, including prostate, breast, lung, and hepatocellular cancer, as potential targets. Importantly, preclinical and clinical research demonstrated that fatostatin, statins, and N-BPs targeting SREBP-2, HMGCR, and FPPS, respectively, alone or in combination with other drugs, have been used for the treatment of different cancers. This review summarizes new insights into the critical role of the SREBP-2-regulated mevalonate pathway for cancer and its potential for targeted cancer therapy.

## Introduction

Sterol regulatory-element binding proteins (SREBPs) were first identified as a subclass of membrane-bound, basic helix-loop-helix leucine zipper (bHLH-LZ) transcription factors which regulate the promoters of genes involved in lipid synthesis and uptake pathways (1–3). In mammals, two genes, *SREBF1* and *SREBF2*, express three major SREBP proteins (SREBP-1a, SREBP-1c, and SREBP-2) with distinct but overlapping lipogenic transcriptional programs (3, 4). Most studies report that SREBP-1a and SREBP-1c primarily regulate fatty acid metabolism and that SREBP-2 is a main regulator of cholesterol metabolism (5–8). Over the past 30 years, the functions of SREBPs have been identified to participate in numerous crucial physiologic processes (9), highlighting metabolic integrators in cellular homeostasis (10, 11). Accumulating evidence has revealed that SREBPs integrate multiple cell signals to control lipogenesis as well as unexpected pathways in type II diabetes, atherosclerosis, and a series of cancers (12, 13).

In particular, multiple SREBP-2-mediated pathways have been extensively studied as attractive potential targets for cancer therapy (14–16). As reported, SREBP-2 binds to the sterol regulatory elements (SREs) in the promoters of its target genes and activates the transcription of mevalonate pathway genes, such as 3-hydroxy-3-methyl-glutaryl-CoA (HMG-CoA) reductase (HMGCR), mevalonate kinase (MVK), and other key enzymes (1). Recent reports found that the mevalonate pathway and its metabolites are essential for cancer growth and malignant progression in a series of cancers, including prostate, breast, lung, and liver cancer (17, 18). Moreover, multiple key pathways, such as the p53 and phosphatidylinositol-3-kinase (PI3K)/Akt signaling pathways, lead to the activation of SREBP-2 to promote tumorigenesis (19–21). Based on the findings above, targeting SREBP-2 and mevalonate pathways has emerged as an encouraging strategy for cancer therapy.

In this review, we first summarized recent advances in the study of SREBP-2 structure, activation, and regulation, followed by SREBP-2, key enzymes of mevalonate pathway, their regulation by various signal pathways or metabolites, and their roles in different cancers. Finally, we focused on the inhibition of the SREBP-2-regulated mevalonate pathway by fatostatin, natural products, statins, or amino-bisphosphonates (N-BPs), alone or in combination with other drugs, as potential therapeutic strategies for various cancers. This review provides new insights into the critical role of SREBP-2-regulated mevalonate metabolism in cancer and its potential as a target for cancer therapy.

## SREBP-2 Structure, Activation, and Regulation

### SREBP-2 Structure

**Figure 1:**
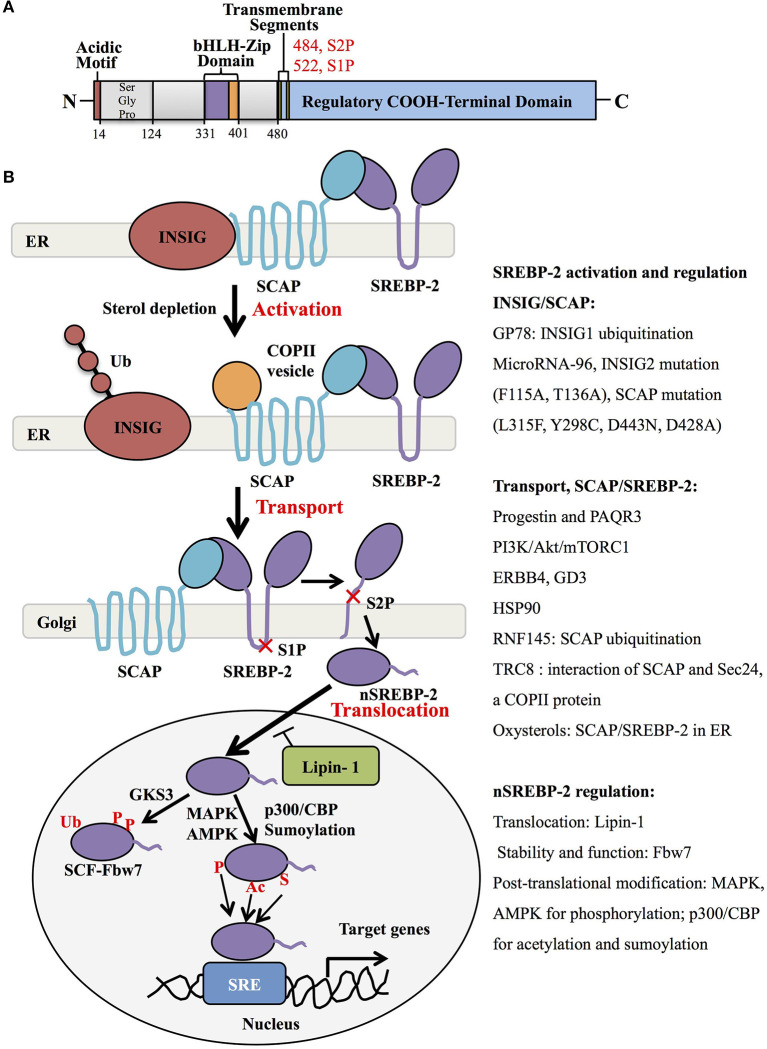
SREBP-2 structure, activation, and regulation. **(A)** SREBP-2 protein consists of three domains, including an NH2-terminal regulatory domain, a middle hydrophilic region, and a COOH-terminal regulatory domain. The NH2-terminal domain contains the bHLH-Zip motif and an acidic transcriptional motif. **(B)** SREBP-2 activation, transport, and translocation. After INSIG dissociation from SCAP by sterol depletion, SREBP-2 translocates to the Golgi apparatus and is cleaved by S1P and S2P proteases to release the NH2-terminal fragment of SREBP-2 (nSREBP-2). nSREBP-2 translocation and stability are regulated by multiple signaling pathways at different levels. ER, endoplasmic reticulum; INSIG, insulin-induced gene protein; SCAP, SREBP cleavage-activation protein; COPII, coatomer protein II; S1P, site-1 protease; S2P, site-2 protease; Ub, ubiquitination; GSK3, glycogen synthase kinase 3; P, phosphorylation; SCF-Fbw7, SKP1-cullin-F-Box protein-F-box and WD repeat domain-containing 7; AMPK, adenosine monophosphate-activated protein kinase; MAPK, mitogen-activated protein kinase; p300/CBP, p300 and cyclic AMP response element-binding protein; Ac, acetylation; S, Sumoylation; SRE, sterol regulatory element; GP78, a membrane-anchored ubiquitin ligase; PAQR3, progestin and adipoQ receptors member 3; ERBB4, Erb-b2 receptor tyrosine kinase 4; GD3, a dominant melanoma ganglioside; HSP90, heat shock protein 90; TRC8, translocation in renal cancer from chromosome 8; RNF145, RNF finger protein 145.

Human SREBP-2, identified by cDNA cloning in 1993, is produced from one gene, *SREBF-2*, 72kb, human chromosome 22q13, as the result of alternative promoter usage and transcription start sites (1). Similar to SREBP-1 structure, SREBP-2 contains 1,141 amino acids and includes an NH_2_-terminal transcription factor domain, a middle hydrophobic region and a COOH-terminal regulatory domain (3). The NH_2_-terminal domain with ~480 amino acids contains the bHLH-Zip motif (DNA binding) and an acidic transcriptional motif (transcriptional activity), which binds co-activator specificity protein 1 (SP1) or nuclear transcription factor Y (NF-Y) to regulate gene expression (22, 23). A middle hydrophobic region of SREBP-2 with approximately 80 amino acids, a membrane-binding region, consists of two hydrophobic membrane-spanning segments separated by a hydrophilic loop, which extends into the lumen of the endoplasmic reticulum (ER). The COOH-terminal regulatory domain contains approximately 590 amino acids responsible for SREBP-2 subcellular localization and translocation (Figure 1A) (24).

### SREBP-2 Activation

Generally, SREBP-2 is synthesized as 125kDa inactive precursors in the ER (9). The COOH-terminal domain of SREBP-2 binds to the WD-repeat domain of SREBP cleavage-activation protein (SCAP), while the NH2-terminal domain of SCAP binds to the ER-resident insulin-induced gene proteins (INSIG), including INSIG1 and INSIG2, to form a complex of INSIG/SCAP/SREBP-2 for maintaining SREBP-2 in the ER (25, 26). When sterol level decreases, SCAP dissociates from INSIGs and facilitates the incorporation of SCAP/SREBP into coatomer protein II (COPII)-coated vesicles, which then transports the complex from the ER to the Golgi apparatus (27, 28). In the Golgi, SREBP-2 is sequentially cleaved by two membrane-bound proteases, site-1 protease (S1P) (29) and site-2 protease (S2P) (30) to release the NH_2_-terminal form of this transcription factor (nuclear SREBP-2, nSREBP-2) (28). This form translocates to the nucleus and binds to the sterol regulatory element (SRE) of target genes, including key enzymes of cholesterol biosynthesis and uptake (4).

Many studies have shown that the INSIG/SCAP/SREBP-2 complex and the transport of SREBP-2 from ER to Golgi are regulated by multiple signaling proteins. When sterols in the ER membrane are high, they bind to loop 1 of SCAP and switch the conformation of SCAP to interact with INSIG protein, which blocks COPII binding and causes the maintenance of the SCAP/SREBP-2 complex in the ER (31) (Figure 1B, upper). Three different mutants within the sterol-sensing domain of SCAP (L315F, Y298C, and D443N) disrupt the interaction of SCAP/INSIG to abolish the sterol-mediated feedback regulation of SREBP processing (32). A recent report showed that heat shock protein 90 (HSP90) stabilized the SCAP/SREBP complex to facilitate SREBP activation (33). Another intrinsic protein encoding an E3 ubiquitin ligase in ER, TRC8 (translocation in renal cancer from chromosome 8), is capable of binding both SREP-2 and SCAP to form a TRC8/SREBP-2/SCAP complex, which hampers the interaction between SCAP and Sec24, a COPII protein, to reduce the cleavage of SREBP-2 (34). Meanwhile, INSIG-1 binds to GP78, a membrane-bound ubiquitin ligase with high affinity, and is then ubiquitinated and rapidly degraded in sterol-depleted cells. However, INSIG-2 lacks interaction with Gp78, which may be related to its slower degradation than INSIG-1 (35, 36). In addition, oxysterols such as 25-hydroxycholesterol bind directly to INSIGs to trigger ER retention of the SCAP/SREBP-2 complex. Mutations at F115A and T136A of the transmembrane helices of INSIG-2 are important for binding to oxysterols and SCAP (37).

For the transporting process, the mutant SCAP with aspartic acid replacement by alanine at 428 (D428A) fails to dissociate from INSIGs and impairs the transportation of SREBP-2 to the Golgi (38). Similar to INSIG-1, INSIG-2 binds SCAP to block the export of SREBPs in the absence of exogenous sterols (25), which is inhibited by microRNA-96 to increase the abundance of active SREBP-2 (39). Furthermore, several signaling proteins were reported to control the transport of SREBP-2. One study showed that Golgi-localized transmembrane protein progestin and adipoQ receptor 3 (PAQR3) interacted with the SCAP/SREBP-2 complex to remain in the Golgi, which was disrupted to reduce cholesterol biosynthesis (40). Another report demonstrated that a RING-finger ubiquitin ligase, RNF finger protein 145, triggered the ubiquitination of SCAP on lysine residues within a cytoplasmic loop, potentially inhibiting the transport of SREBP-2 to Golgi and subsequent SREBP-2 processing (41). Additionally, the PI3K/Akt/mTORC1 pathway is involved in SREBP-2 transport to the Golgi, contributing to SREBP-2 activation (42, 43), which can be activated by neuregulin-activated ERBB4 and melanoma antigen ganglioside GD3 (19, 44). Collectively, the INSIG/SCAP/SREBP-2 complex and SREBP-2 transportation from ER to Golgi are regulated by multiple signaling molecules, as summarized in Figure 1B.

### SREBP-2 Regulation at Different Levels

After the cleavage of full-length SREBP-2 by S1P and S2P in the Golgi, nSREBP-2 can translocate to the nucleus and be regulated at protein expression and transcription levels. The nutrient and growth factor-responsive kinase mTOR complex 1 (mTORC1) causes Lipin-1, a phosphatidic acid phosphatase, to reside in the cytoplasm, which increases the expression of nSREBP-2 protein (45, 46). mTORC1 can also suppress cholesterol delivery to lysosomes through the inhibition of autophagy and the maintenance of endosomal recycling, which reduces the level of cholesterol in ER to activate SREBP-2 (47). A nuclear receptor protein, peroxisome proliferator-activated receptor (PPAR) α in in rat liver cells, leads to a decrease of nSREBP-2 to lower cholesterol concentration (48). In addition, the stability and function of nuclear SREBP-2 are negatively regulated by a substrate receptor of the SCF ubiquitin ligase complex, Fbw7, through ubiquitination and proteasome-mediated degradation in a phosphorylation-dependent manner (49).

Importantly, the transcriptional activity of nSREBP-2 is also modulated by various post-translational modifications, including phosphorylation, acetylation, and sumoylation. For the phosphorylated regulation, insulin-activated Erk-mitogen-activated protein kinase (MAPK) increases SREBP-2 activity by phosphorylation at serine 432 and 455 (50). Glycogen synthase kinase 3 directly phosphorylates Ser443 on SREBP-2 to mediate Fbw7-induced ubiquitination and degradation of nSREBP-2 (49). A synthetic polyphenol, S17834, can promote AMP-activated protein kinase (AMPK) activation to decrease SREBP-2 transcription via its phosphorylation site on SREBP-2 (51). Aside from phosphorylation, histone acetyltransferase p300/CREB-binding protein (CBP) can bind and acetylate the N-terminus of SREBP-2 to enhance its expression and transcriptional activity (52), while sirtuin-1 (SIRT1) deacetylates SREBP-2 to decrease the abundance of SREBP-2 in the nucleus (53). Additionally, nSREBP-2 at Lys464 is also modified by sumoylation to decrease transcriptional activity (54). Taken together, SREBP-2 stability and activation are regulated by a series of key molecules and signaling pathways, which hold promise for understanding the role of SREBP-2 in physiological and pathological procedures.

## The SREBP-2-Regulated Mevalonate Pathway

**Figure 2:**
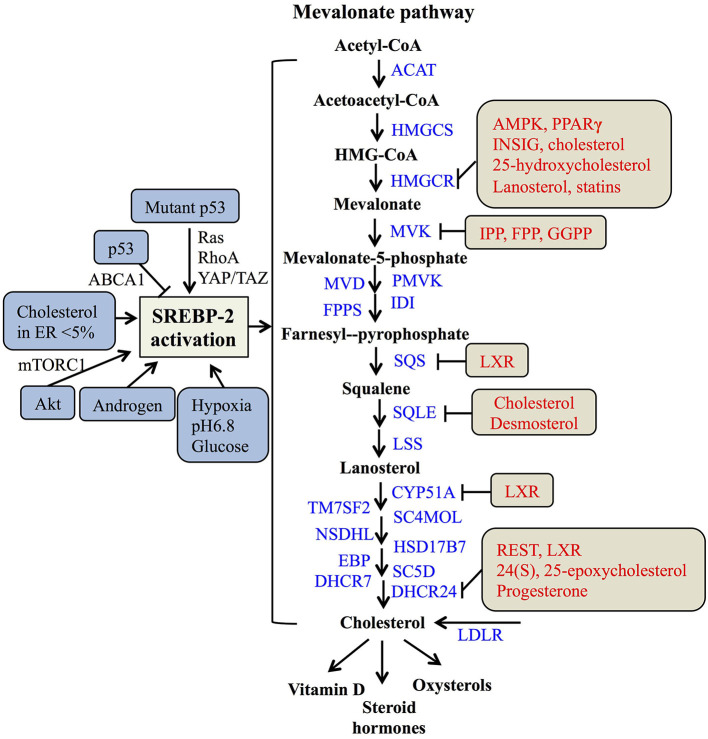
The SREBP-regulated mevalonate pathway and its regulation. Schematic representation summarizing the SREBP-2-regulated mevalonate pathway and key enzymes for synthesis from acetyl-CoA to cholesterol and its products. Multiple signaling pathways such as p53, Akt, and androgen can regulate SREBP-2 activation. Several regulatory feedback mechanisms exist for different enzymes by various signals and mevalonate metabolites, such as cholesterol, IPP (Isopentenylpyrophosphate) and FPP (farnesylpyrophosphate). ACAT, acyl CoA-cholesterol acyltransferase; HMGCSm HMG-CoA synthase; HMGCR, HMG-CoA reductase; MVK, mevalonate kinase; MVD, HMG-CoA synthase; PMVK, phosphomevalonate kinase; IDI, isopentenyl diphosphate isomerase; FPPS, farnesylpyrophosphate synthase; SQS, squalene synthase; SQLE, squalene epoxidase; LSS, lanosterol synthase; CYP51A, lanosterol-14α demethylase; TM7SF2, steroid 14 reductase; SC4MOL, 4 methyl sterol oxidase; NSDHL, C3 sterol dehydrogenase; HSD17B7, 3-ketoreductase, EBP, phenylalkylamine Ca^2+^ antagonist binding protein; SC5D, sterol-C5-desaturase; DHCR7, 7-dehydrocholesterol reductase; DHCR24, 24-dihydrocholesterol reductase; LDLR, low-density lipoprotein receptors; GGPP, geranylgeranylpyrophosphate; AMPK, adenosine monophosphate-activated protein kinase; PPARγ, peroxisome proliferators-activated receptor γ; INSIG, insulin-induced gene protein; LXR, Liver X receptor; REST, RE1-silencing transcription factor.

In the nucleus, nSREBP-2 binds to SREs in the promoter of target genes to activate the gene expression of most of the enzymes involved in the mevalonate pathway, including HMGCR, MVK, squalene synthase (SQS) (55), and 24-dihydrocholesterol reductase (DHCR24) (56), as well as increasing the expression of low-density lipoprotein receptors (LDLR) for exogenous cholesterol uptake (8, 57). For the mevalonate pathway, two molecules of acetyl-CoA from glucose metabolism or fatty acid degradation form acetoacetyl-CoA by acetoacetyl-CoA thiolase. In the presence of HMG-CoA synthase (HMGCS), acetyl-CoA and acetoacetyl-CoA form HMG-CoA, which is converted to mevalonate by HMGCR (58). Then, the mevalonate is phosphorylated sequentially to 5-phosphomevalonate by mevalonate kinase (MK) and to 5-pyrophosphomevalonate by phosphomevalonate kinase (PMK), which is further synthesized to isopentenylpyrophosphate (IPP) by mevalonate diphosphate decarboxylase (59). Furthermore, IPP and its isomer, dimethylallyl pyrophosphate (DMPP), can form geranyl pyrophosphate (GPP) by farnesylpyrophosphate synthase (FPPS, FDPS), which is condensed with another IPP to yield farnesylpyrophosphate (FPP). By the action of SQS, FPP is converted to squalene (60), which is converted sequentially to monooxidaosqualene (MOS) and lanosterol by squalene monooxygenase (SM) and lanosterol synthase, respectively (18). Lastly, lanosterol is further metabolized to cholesterol by 19 enzymes, including CYP51A (lanosterol-14α demethylase), TM7SF2 (steroid 14 reductase), SC4MOL (4 methyl sterol oxidase), NSDHL (C3 sterol dehydrogenase), HSD17B7 (3-ketoreductase), EBP (phenylalkylamine Ca^2+^ antagonist binding protein), SC5D (sterol-C5-desaturase), 7-dehydrocholesterol reductase (DHCR7), and DHCR24 (17, 56, 61). As reported, cholesterol plays a crucial role in maintaining the structure and function of cellular membranes and is also a precursor of steroid hormones and vitamin D (62). Collectively, SREBP-2 controls cholesterol biosynthesis by regulating mevalonate metabolism enzymes (Figure 2).

As many studies reported, SREBP-2 activation and pathways are regulated by multiple signals. A major tumor suppressor, p53, can block activation of SREBP-2 to decrease the transcription of mevalonate pathway genes through transcriptional up-regulation of the ATP-binding cassette (ABC) transporter A1 (ABCA1) gene, which mediates tumor suppression (21). On the other hand, SREBP-2 can increase the generation of oxysterol ligands for liver X receptors (LXRs) to positively regulate ABCA1 gene transcription (63). LXRs play a potential role in maintaining cholesterol homeostasis through promoting cholesterol efflux and suppressing *de novo* synthesis and uptake (64, 65). Mutant p53 is recruited to the promoters of genes encoding mevalonate pathway enzymes by binding to the SREBP-2, which subsequently increases the activities of oncogenic pathways such as Ras, RhoA (66), and YAP/TAZ (67) to promote cancer progression (20, 68). Apart from p53, protein kinase B (Akt) acutely activates SREBP-2 (43) to induce the expression of genes involved in cholesterol synthesis, which contributes to tumor development (19, 69). In addition, tumor microenvironments such hypoxia, extracellular pH, and nutrient levels also play critical roles in the regulation of SREBP-2 activation. Hypoxia inducible factor-1α is able to increase the activity of HMGCR by the translocation of SREBP-2 to the nucleus (70). Acidic extracellular pH (pH 6.8) triggers nuclear translocation of SREBP-2 to target acyl-CoA synthase short-chain family member 2 for maintaining overall survival of cancer patients (71). As a kind of nutrient, glucose promotes SCAP/SREBP complex trafficking from the ER to the Golgi and subsequent SREBP activation via N-glycosylation of SCAP (72). When cholesterol in the ER falls below 5% of total ER lipids, the cleavage of SREBP-2 is activated (31). A steroid hormone, androgen, can induce SREBP-2 activation in normal physiological or pathological conditions, such as prostate cancer (73, 74). Taken together, either multiple signaling pathways or cellular nutrient levels can regulate SREBP-2 activation to control the mevalonate pathway (Figure 2).

Meanwhile, the enzymes participating in the mevalonate pathway, such as HMGCR, MVK, SQS, and DHCR24, are regulated by various molecules or the metabolites from mevalonate metabolism. Both the phosphorylation by AMP-activated protein kinase (AMPK) and dephosphorylation by protein phosphatase 2A regulate HMGCR activity (75, 76). The binding of INSIG on the sterol-sensing domain can lead to the ubiquitination and degradation of HMGCR (77, 78). Interestingly, PPAR**γ** can regulate multiple pathways, including decreasing the expressions of SREBP-2 and HMGCR and increasing the expression of LXRα to reduce cholesterol levels. Considering the role of LXRα on cholesterol efflux, the expression of ABC transporter G5 or G8 is increased by the PPAR**γ**-LXRα pathway or their individual dependence, which needs to be further clarified (79). Two key enzymes of the post-squalene pathway, SQS and CYP51A are directly repressed by LXRα via negative binding with LXR DNA response elements (80). Moreover, DHCR24 as a final enzyme for cholesterol biosynthesis is regulated by RE1-silencing transcription factor, REST and LXRα through the binding of its promoter at the transcriptional level (56, 81). These findings suggest that LXRα plays an important role in regulating several enzymes of the mevalonate pathway, such as SQS, CYP51A, and DHCR24.

Additionally, key metabolites also can modulate metabolic enzymes of the SREBP-2-reguated mevalonate pathway. Cholesterol and 25-hydroxycholesterol can regulate HMGCR by increasing its alternative splicing (82). Mevalonate and certain of its derivatives such as dioxidolanosterol and geraniol regulate HMGCR mRNA translation or polysome distribution to reduce its synthesis and translation (83). Lanosterol and other C4-dimethylated sterol intermediates may regulate both HMGCR degradation and SREBP-2 cleavage (84). For geranylgeranyle diphosphate (GGPP), FPP and IPP, these intermediates post-transcriptionally inhibit MVK activity by negative feedback responses (85). Other metabolites, such as phytosterols, 24(S), 25-epoxycholesterol (24,25-EC) and steroid hormones (progesterone) can directly inhibit DHCR24 activity at the post-translational level (86–88). Overall, SREBP-2 and the enzymes for cholesterol biosynthesis, such as HMGCR, MVK, SQS, and DHCR24, can be regulated by various signaling pathways and mevalonate pathway metabolites at the transcriptional and post-translational levels (Figure 2).

## SREBP-2 Signaling and the Enzymes From the Mevalonate Pathway in the Cancer Context

Reprogramming of lipid metabolism occurs in a variety of cancers and contributes to rapid tumor growth, which is regulated by SREBPs (89). SREBP-2 is markedly upregulated in various cancers, including prostate (14, 90), breast (15), and hepatocellular cancer (91). Moreover, SREBP-2-mediated mevalonate metabolism drives epithelial to mesenchymal transition (EMT) and supports cancer stemness, and has been suggested as a potential target for cancer treatment (17, 18, 92).

### Prostate Cancer

Lipid synthesis and uptake are significantly elevated in prostate cancer (PCa) as important energy resources to support tumor growth and progression (93, 94). As is well-known, androgens bind to and activate the androgen receptor (AR) to maintain the survival and proliferation of PCa (95). Androgen-induced activation of SREBPs occurs not only under normal physiological conditions but also in the setting of steroid-regulated cancers (74, 96). Androgens markedly stimulate the expression of SCAP (97) and cause a switch in the isoform expression of INSIG, which play a pivotal role in the lipogenic effects of androgen in PCa (73). Meanwhile, dihydrotestosterone or R1881 marginally up-regulates the mRNA and protein levels of SREBP-2, which induces the expression of multiple genes encoding enzymes involved in cholesterol biosynthesis, including HMGCS, HMGCR, FPPS in PCa cells (98, 99). A recent report shows that an aberrant SREBP-dependent lipogenic program promotes PCa metastasis with double-null PML and PTEN (100). During the progression to androgen independence, nuclear SREBP-2 protein expression underwent a 3-fold increase in a PCa xenograft model (90). In addition, SREBP-2 expression is elevated in advanced pathologic grade and metastatic PCa and significantly associated with poor clinical outcomes. SREBP-2 promotes PCa cell growth, stemness and metastasis through transcriptional c-Myc activation mediated by direct interaction with a SREBP-2-binding element in the 5′-flanking c-Myc promoter region (14).

Key enzymes for mevalonate pathway such as HMGCS1, HMGCR, FPPS, and SQS also play important roles in PCa malignant progression. HMGCS1 and HMGCR are overexpressed in stroma of early stage PCa (101). Moreover, enzalutamide-resistant PCa cell lines express elevated HMGCR, and are more sensitive to statins, HMGCR inhibitors (102). FPPS is associated with increasing Gleason scores, PTEN functionally deficient status, and poor survival in PCa through modulation of the small GTPases/Akt axis (103, 104). SQS at rs2645429 is significantly associated with PCa risk and aggressive phenotypes (105). Taken together, SREBP-2 and key enzymes for the mevalonate pathway are potential targets for PCa treatment.

### Breast Cancer

In breast cancer, CtBP expression negatively correlates with SREBP-2 and HMGCR expressions. CtBP can form a complex with ZEB1 to transcriptionally repress SREBP-2 expression and activate TGF-β signaling, which maintains intracellular cholesterol homeostasis in breast cancer (106). TP53 mutation correlates with elevated expression of a subset of mevalonate pathway genes in breast cancer patients. The levels of genes such as HMGCR, FPPS, SQS, and DHCR7 are positively associated with the risk of breast cancer. The functional interaction with SREBP-2 is critical for mutant p53-mediated up-regulation of the mevalonate pathway genes (20). Oncogenic PI3K (H1047R) or K-Ras (G12V) can induce *de novo* lipogenesis through convergent activation of mTORC1 to promote aberrant growth and proliferation of breast cancer, which is mediated by the activation of SREBP-2 or SREBP-1 (107). In addition, SREBP-2 is highly expressed in breast cancer tissues and correlated with a poor prognosis (15). SREBP-2 expression is increased during the early stages of osteoclast formation under the control of the RANKL/cAMP-CREB signaling cascade, which induces the expressions of NFATc1 and matrix metalloproteinase, thus contributing to breast cancer-induced osteolysis (15).

For patients with HER2^+^ metastatic breast cancer, dual targeted therapy with a tyrosine kinase inhibitor, lapatinib or its combination with an anti-HER2 monoclonal antibody, trastuzumab can significantly improve pathological complete response and overall survival (108). However, lapatinib and its combination with trastuzumab lead to the resistance of breast cancer cells to HER2-targeted therapy, which has been a clinical challenge (109). The mevalonate pathway has been considered as a new potential target for overcoming this acquired anti-HER2 treatment resistance, which may be mediated by activating the mTORC1-mediated YAP/TAZ pathway (110). Rate-limiting enzyme studies found that high levels of HMGCR are correlated with breast cancer risk (111) and poor survival (112, 113). Cholesterol is also implicated as a breast cancer risk factor and promotes breast tumor growth and metastasis (114). Another metabolite, 27-hydroxycholesterol, can increase the proliferation of estrogen receptor (ER)-positive breast cancer through the activation of ER and LXR (115). Therefore, inhibition of the SREBP-2-mediated mevalonate pathway has been recognized as a potential therapeutic approach for breast cancer.

### Lung Cancer

The single-nucleotide polymorphism of HMGCR, rs12916, is associated with the subgroups of attained age for lung cancer (111) and the C allele of the SQS rs2645429 polymorphism gene can be a risk factor for non-small cell lung cancer (NSCLC) (116). Three key enzymes of the mevalonate pathway, FPPS, SQS and GGPPS, are also associated with stage and metastasis of NSCLC (117–119). Of these enzymes, SQS is increased in invasive lung cancer cells and in the tumor regions of lung cancer specimens, and significantly associated with metastasis and poor prognosis by enhancing NF-κB-mediated up-regulation of matrix metallopeptidase-1 (117) or modulating extracellular signal-regulated kinase (ERK) signaling (120). FPPS plays an important role in promoting cell invasion and EMT through the RhoA/ROCK1 pathway (118). Although GGPPS knockdown has no effect on lung adenocarcinoma cell proliferation and apoptosis, it significantly inhibits invasion and migration by regulating EMT (119). Overall, several enzymes from the mevalonate pathway as mentioned above have been identified as potential targets for treating lung cancer (121).

### Hepatocellular Carcinoma

New studies reveal that several key molecules, such as p53 and fatty acid synthase (FASN), can activate SREBP-2 to promote cholesterol accumulation for maintaining the progression of hepatocellular carcinoma (HCC). In HCC, p53 tumor suppressor can induce the expression of MVA pathway enzymes through the accumulation and stabilization of mature SREBP-2 by transcriptionally inducing ABCA1, a cholesterol transporter gene. Like p53 loss, the ablation of ABCA1 promotes murine liver tumorigenesis and is associated with increased SREBP-2 maturation (21). In contrast to p53, a p53 activator, haplo-insufficient tumor suppressor ASPP2, can interact with SREBP-2 in the nucleus and negatively regulates the mevalonate pathway to mediate the inhibition of HCC tumor growth (122). Moreover, overexpression of Staphylococcal nuclease and tudor domain containing-1 (SND-1) in HCC results in the accumulation of cellular cholesteryl esters due to the altered activation of SREBP-2 (123). Interestingly, SREBP-2 also binds to specific sites in SND-1 promoter to induce its transcription, which contributes to lipid metabolism reprogramming in HCC (91). This suggests that there is a complex for the interaction of SND-1and SREBP-2 in the lipid reprogramming of HCC, which needs to be clarified. Another molecule, FASN, contributes to *de novo* fatty acid synthesis in a murine HCC model induced by Pten loss and c-Met overexpression. Compared with the control group, genes such as HMGCR involved in cholesterol biosynthesis were obviously upregulated in HCC in FASN knockout mice, related to the promotion of nuclear SREBP-2 (124). Reportedly, the inhibition of FASN ubiquitination and disruption of the SREBP-1/SREBP-2 degradation complexes may be potential molecular mechanisms of Akt-induced lipogenesis and HCC tumor development in mice (69). In addition, Forkhead Box M1 has a positive correlation with SREBP-2 or HMGCR in HCC tissues, which links the mevalonate pathway through protein geranylgeranylation as novel targets (125). Based on the findings above, targeting the SREBP-2-mediated mevalonate pathway seems to have potential as a strategy for HCC treatment.

### Other Cancers

Similarly, SREBP-2 and its regulated mevalonate pathways also participate in other cancers. In esophageal squamous cell carcinoma, SREBP-2 is upregulated in clinical samples and promotes cell growth, migration and colony formation, which may be mediated by interaction with c-Myc to increase HMGCR expression (16). In renal carcinoma, kruppel-like factor 6 (KLF6) activates mTOR signaling and its downstream lipid metabolism regulator, SREBP-2 to enhance tumor growth (126). In pancreatic cancer, the novel small nucleolar RNA host gene 16 directly regulates the miR-195/SREBP-2 axis to promote lipogenesis and accelerate tumor progression (127). Furthermore, increasing cellular cholesterol can drive intestinal stem cell proliferation and tumorigenesis through the activation of nuclear SREBP-2 (128). Also, SQS is frequently mutated and dysregulated in the liver metastatic cohort of colorectal cancer (129). The final enzyme of the cholesterol pathway, DHCR24, is significantly elevated and associated with advanced clinical stage and overall survival in bladder and endometrial cancer, which is mediated by several oncogenesis-associated biological processes (130, 131). Collectively, these findings in different cancers indicate that the SREBP-2-regulated mevalonate pathway significantly participates in tumor growth and metastasis and may be an attractive target in a variety of malignancies (Table 1).

**Table 1:** The roles and molecular mechanisms of the SREBP-2-regulated mevalonate pathway in different cancers.

**Cancer type**	**Targets**	**Molecular mechanism from the findings**
Prostate cancer	SREBP-1, SREBP-2, HMGCS, HMGCR, FPPS	Androgen induces the activation of SREBPs and the expression of multiple enzyme genes, including HMGCS, HMGCR and FPPS in normal physiological conditions and steroid-regulated cancers (74, 96)
	SCAP, INSIG	Androgen stimulates SCAP expression and causes a switch in INSIG isoform for lipogenesis (73, 97)
	SREBP-2	Induced by a 3-fold increase during the progression to androgen independence (90)
	SREBP-2	Elevated in advanced pathologic grade and metastasis of prostate cancer and significantly associated with poor clinical outcomes (14)
	SREBP-2	Promotes cancer cell growth, stemness and metastasis through transcriptional c-Myc activation (14)
	HMGCS1, HMGCR	Overexpressed in stroma of early stage PCa (101)
	HMGCR	Elevated in enzalutamide-resistant cancer and more sensitive to statins (102)
	FPPS	Associated with increasing Gleason score and poor survival through modulation of small GTPase/Akt axis (103, 104)
	SQS	The allele at rs2645429 is significantly associated with cancer risk and aggressive phenotypes (105)
Breast cancer	SREBP-2	CtBP can form a complex with ZEB1 to transcriptionally repress SREBP-2 expression and activate TGF-β signaling (106)
	SREBP-2, HMGCR, FPPS, SQS, DHCR7	TP53 mutation upregulates with the mevalonate pathway genes, HMGCR, FPPS, SQS, and DHCR7 through interaction with SREBP-2 (20)
	SREBPs	PI3K or K-Ras can induce mTORC1 signaling to promote cancer growth through SREBP-2 or SREBP-1 activation (107)
	SREBP-2	Highly expressed in cancer tissues and correlated with a poor prognosis (15)
	SREBP-2	Increased during the early stages of osteoclast formation under the control of the RANKL/cAMP-CREB signaling and induced the expressions of NFATc1 and matrix metalloproteinases for cancer-induced osteolysis (15)
	HMGCR	Correlated with the cancer risk and poor survival (111–113)
	Cholesterol	Implicated as a cancer, tumor growth and metastasis risk factor (114)
	27-hydroxycholesterol	Increases the proliferation of estrogen receptor (ER)-positive breast cancer through the activation of ER and LXR (115)
Lung cancer	HMGCR	The allele at rs12916 is significantly associated with the attained age for cancer patients (111)
	SQS	The allele at rs2645429 is a risk factor for non-small cell lung cancer (NSCLC) (116)
	SQS	Associated with the metastasis and poor prognosis by regulating NF-κB-mediated the up-regulation of matrix metallopeptidase-1 or extracellular signal-regulated kinase signaling (117, 120)
	FPPS	Promotes cell invasion and epithelial mesenchymal transition (EMT) through the RhoA/ROCK1 pathway (118)
	GGPPSS	Increases cancer invasion and migration by regulating EMT (119)
Hepatocellular carcinoma	SREBP-2	p53 induces the accumulation and stabilization of mature SREBP-2 by transcriptional ABCA1 induction (21)
	SREBP-2	ASPP2, a p53 activator interacts with SREBP-2 in the nucleus to negatively affect the mevalonate pathway (122)
	SREBP-2	Staphylococcal nuclease and tudor domain containing-1 (SND-1) results in the accumulation of cholesteryl esters through the activation of SREBP-2 (123)
	SREBP-2	Binds to specific sites in SND-1 promoter to contribute lipid metabolism reprogramming (91)
	SREBP-2, HMGCR	Fatty acid synthase ablation promotes nuclear localization of SREBP-2 and increases HMGCR expression to maintain carcinogenesis (124)
	SREBP-2, HMGCR	Forkhead Box M1 has a positive correlation with SREBP-2 or HMGCR in hepatocellular carcinoma through protein geranylgeranylation (125)
Esophageal squamous cell carcinoma	SREBP-2	Promotes cell growth, migration and colony formation through interaction with c-Myc; SREBP-2 is upregulated in clinical samples (16)
Renal carcinoma	SREBP-2	Kruppel-like factor 6 activates mTOR-SREBP-2 to enhance tumor growth (126)
Pancreatic cancer	SREBP-2	Small nucleolar RNA host gene 16 directly regulates the miR-195/SREBP-2 axis to promote cancer progression (127)
Colorectal cancer	SREBP-2	Increasing cellular cholesterol drives intestinal stem cell proliferation and tumorigenesis through SREBP-2 expression (128)
	SQS	Frequently mutated and dysregulated in liver metastasis (129)
Bladder and endometrial cancer	DHCR24	Significantly elevated and associated with advanced clinical stage and overall survival (130, 131)

## Targeting the SREBP-2-Regulated Mevalonate Pathway For Cancer Therapy

Based on the above reports, we choose SREBP-2, HMGCR, and FPPS as potential targets for cancer therapy and summarized the findings so far regarding several inhibitors or miRNAs used to address these targets in preclinical and clinical studies.

### Targeting SREBP-2 for Cancer Therapy

As reported, SREBPs inhibition by small molecules such as fatostatin, natural products, and microRNAs such as miR-185, miR-342, and miR-33a have been extensively found to exert multiple anti-tumor effects in various cancers by reducing mevalonate metabolic dysfunction (132–136). Fatostatin, a non-sterol diarylthazole derivative, was first reported to inhibit insulin-induced adipogenesis and reduce body weight by blocking nuclear translocation of SREBPs in obese mice (137, 138). Fatostatin has been used for treating prostate (133), breast (139), and endometrial cancers (140). Mechanistically, fatostatin directly binds SCAP and blocks its transport from ER to Golgi apparatus, then inhibits the activation of SREBPs (138). A recent study also showed that fatostatin inhibits cell proliferation through a SCAP-independent mechanism (141). In PCa, *in vitro* and *in vivo* studies reveal that fatostatin suppresses cell proliferation and induces apoptosis through blockade of SREBP-regulated metabolic pathways (133), similar to the findings in endometrial carcinoma (140). The combination of fatostatin with docetaxel significantly increases proliferation inhibition and apoptosis induction in metastatic PCa harboring p53 mutations, compared with fatostatin alone (142). Moreover, fatostatin also inhibits mitotic microtubule spindle assembly and cell division in aggressive cancers in addition to the inhibition of SREBP activity (136). Fatostatin also causes lipid accumulation as a response to endoplasmic reticulum stress rather than the inhibition of SREBP-mediated lipogenesis in ER^+^ breast cancer cells (139). These studies suggest that the antitumor effects of fatostation are multiple and dependent on cancer type.

Recent studies indicate that natural products can directly target SREBP-2 to inhibit the expression of key enzymes for the mevalonate pathway, to reduce tumor growth. Tocotrienol, a minor form of vitamin E, can degrade mature SREBP-2 without affecting LXR activity to maintain cholesterol homoeostasis in PCa (143). In glioma, artesunate, initially developed as an anti-malaria drug, effectively inhibits cancer cell growth and distant metastasis, and further induces cell senescence by regulating the nuclear localization of SREBP-2 and the expression of HMGCR (144). As an anthraquinone derived from many plants, emodin inhibits SREBP-2 transcriptional activity to suppress cholesterol metabolism and Akt signaling, which sensitizes HCC cells to the anti-cancer effect of sorafenib *in vitro* and in xenograft models (145). Surprisingly, ursolic acid as a natural pentacyclic terpenoid activates SREBP-2 and increases the expression of cholesterol biosynthesis-related enzymes to induce cell cycle arrest and apoptosis in HCC cells (146). Additionally, archazolid B leads to the accumulation of free cholesterol and drastic disturbance in cholesterol homeostasis, which can activate nuclear SREBP-2 expression and up-regulate HMGCR for killing bladder cancer cells (147).

Some miRNAs, such as miR-98 and miR-33a, have been found to play critical roles in cholesterol metabolism by targeting SREBP-2 (134, 148, 149). Our previous study shows that miR-185 and miR-342 not only significantly block SREBP-2-mediated cholesterogenesis, but also inhibit SREBP-1-mediated lipogenesis in PCa (132). Another miRNA, miR-33a, an intronic miRNA located within the SREBP-2 gene, inhibits EMT targeting of Twist1 to block invasion and metastasis in NSCLC (135). According to present studies, searching for miRNAs directly and specifically targeting SREBP-2 could be a future direction for new cancer treatment strategies. Table 2 summarizes current SREBP-2 targeting by small molecules or miRNAs.

**Table 2:** Preclinical findings for targeting the SREBP-2-regulated mevalonate pathway in different cancers.

**Treatment**	**Targets**	**Cancer type**	**Molecular mechanism**
Fatostatin	SREBP-regulated metabolic pathway	Prostate cancer	Inhibits cell proliferation, colony formation, invasion and migration and causes G2/M cell cycle arrest and apoptosis *in vitro* and *in vivo* (133)
Fatostatin+ docetaxel	SREBP-regulated metabolic pathway	Prostate cancer	Results in greater proliferation inhibition and apoptosis induction in metastatic prostate cancer harboring p53 mutations, compared with fatostatin alone (142)
Fatostatin	SREBP-regulated metabolic pathway	Endometrial carcinoma	Inhibits cell viability, invasive and migratory capacities, and induces cell cycle arrest at the G2/M phase and stimulates caspase-mediated apoptosis (140)
Fatostatin	SREBP activity	Glioma, colorectal cancer, and others	Inhibits SREBP activity and mitotic microtubule spindle assembly and cell division (136)
Tocotrienol	SREBP-2	Prostate cancer	Degrades mature SREBP-2 and has no effect on LXR activity (143)
Artesunate	SREBP-2	Glioma	Inhibits cell growth, distant metastasis and induces cell senescence by regulating SREBP-2 nuclear localization and HMGCR expression (144)
Emodin	SREBP-2	Hepatocellular carcinoma	Inhibits SREBP-2 transcriptional activity to suppress cholesterol metabolism and Akt signaling (145)
Ursolic acid	SREBP-2	Hepatocellular carcinoma	Activates SREBP-2 and increases the expression of cholesterol biosynthesis-related enzymes to induce cell cycle arrest and apoptosis (146)
Archazolid B miRNA-185/342 miRNA-33a	SREBP-2 SREBP-regulated metabolic pathway SREBP-2	Bladder cancer Prostate cancer Non-small cell lung cancer	Activates nuclear SREBP-2 expression and up-regulates HMGCR for killing bladder cancer cells (147) Blocks SREBP-2-mediated cholestergenesis, and inhibits SREBP-1-mediated lipogenesis (132) Inhibits EMT targeting of Twist1 to block tumor progression (135)
Simvastatin	HMGCR	Prostate cancer	Inhibits Akt activity, cell migration and colony formation (150)
Simvastatin, fluvastatin	HMGCR	Prostate cancer	Inhibits cell proliferation and induces apoptosis via the downregulation of Akt/Foxo1 phosphorylation (151)
Simvastatin	HMGCR	Prostate cancer	Overcomes enzalutamide resistance by inhibiting mTOR-mediated AR degradation (102)
Atorvastatin	HMGCR	Breast cancer	Suppresses cancer proliferation, EMT and distant metastasis and induces autophagy by PTEN/Akt and Ras homolog family member B pathways (152–154)
Atorvastatin, lovastatin, simvastatin	HMGCR	Breast cancer (stem cells)	Significantly alters a shared cluster of 37 genes, including Hippo, Notch and Wnt pathways and holds back the EMT process (155)
Simvastatin	HMGCR	Breast cancer	Induces cell death through the deactivation of PI3K/Akt and MAPK/Erk signals (156)
Simvastatin	HMGCR	Triple negative breast cancer	Prevents cancer proliferation and metastasis through Foxo3a or heat shock protein 90 (157, 158)
Pitavastatin	HMGCR	Breast cancer	Slows bone metastasis and reduces urine-derived volatile organic compounds through the mevalonate pathway (159)
Atovastatin	HMGCR	Lung cancer	Inhibits TGF-β1-induced EMT by attenuating the upregulation of SphK1 (160)
Lovastatin	HMGCR	Lung cancer	Elicits cell apoptosis via a COX-2/PPARγ-dependent pathway (161)
Simvastatin	HMGCR	Lung cancer	Down-regulates TGF-βRII expression and inhibits proliferation via Erk (162)
Fluvastatin	HMGCR	Lung cancer	Inhibits bone metastasis and the releases of RANKL, IL-6 and other factors through autophagy induction and osteoclastogenesis (163–165)
Simvastatin	HMGCR	Hepatocellular carcinoma	Induces G_0_/G_1_ arrest by regulating p21 and p27, activating AMPK, and inhibiting STAT3-Skp2 axis (166)
Simvastatin, fluvastatin	HMGCR	Hepatocellular carcinoma	Attenuates cell proliferative ability via TAZ (167)
Simvastatin	HMGCR	Hepatocellular carcinoma	Induces growth inhibition and apoptosis via upregulation of Notch1 (168)
Simvastatin	HMGCR	Hepatocellular carcinoma	Modulates the adhesion and growth via decrease of integrin expression and ROCK (169)
Fluvastatin	HMGCR	Renal cell carcinoma	Has potent anti-cancer effects through suppression of the Akt/mTOR signaling cascade (170)
Fluvastatin	HMGCR	Lymphoma	Induces apoptosis by promoting ROS generation and regulating Akt, Erk and p38 signaling pathways (171)
Atovastatin + celecoxib	HMGCR	Prostate cancer	Inhibits the progression of androgen dependence to androgen independence (172)
Lovastatin + doxorubicin	HMGCR	Ovarian cancer	Induces apoptosis by blocking HMG-CoA reductase activity and inhibiting P-glycoprotein (173)
Statins + venetoclax	HMGCR	Leukemia and lymphoma	Enhances the proapoptotic activity of venetoclax by blocking mevalonate production and upregulating PUMA (174)
Simvastatin + Metformin Simvastatin + AZD6244	HMGCR HMGCR	Endometrial carcinoma Pancreatic and Prostate cancer	Synergistically inhibits growth and induces apoptosis by upregulating AMPK phosphorylation and downregulating S6 phosphorylation (175) Synergize to accumulate ROS production and cause apoptosis by targeting the compensatory xCT cystine importer (176)
Zoledronic acid	FPPS	Prostate cancer	Induces apoptosis through down-regulation of survivin (177)
Zoledronic acid	FPPS	Prostate cancer	Inhibits the RhoA-mediated amoeboid motility and impedes metastatic lung colonization (178)
Zoledronic acid	FPPS	Prostate cancer (stem cells)	Facilitates the intrinsic pathway of apoptosis to overcome chemoresistance (179)
Zoledronic acid	FPPS	Prostate cancer	Markedly induces autophagosome formation (180)
Zoledronic acid	FPPS	Prostate cancer	Inhibits protein prenylation (181)
Zoledronic acid	FPPS	Breast cancer	Significantly reduces the expression of cancer cell factors such as CCL2 and IDO to suppress regulatory T-cell function (182)
Zoledronic acid nanoparticle	FPPS	Breast cancer	Restores doxorubicin cytotoxic efficacy against chemo-immunoresistant tumors by reducing metabolic flux and also lowers the activity of Ras/Erk1/2-HIF-1α axis to maintain cell death and immunosuppression (183)
Zoledronic acid	FPPS	Lung cancer	Causes arrest at S/G_2_/M phase with increases of cyclins and cyclin-related regulatory proteins, such as Ras (184)
Zoledronic acid	FPPS	Lung cancer	Inhibits the prenylations of Ras and Rap1A (185)
Zoledronic acid	FPPS	Hepatocellular carcinoma	Inhibits the translocation of Ras and Rho A to reduce cell growth and prevents progression to bone metastatic lesions (186)
Zoledronic acid	FPPS	Prostate cancer, primary effusion lymphoma	Reverts M2 macrophages to M1 phenotype for producing IFN-γ and activates the Vγ9Vδ2 T cells to suppress tumorigenesis through the immune modulation (187, 188)
YM529	FPPS	Prostate cancer	CXCR-4-induced invasion (189)
YM529	FPPS	Non-small cell lung cancer	Down-regulation of Erk1/2 phosphorylation (190)
YM529	FPPS	Bladder cancer	Inhibition of Rap1A prenylation (191)
Zoledronic acid + docetaxel	FPPS	Prostate cancer	The combination produces the greatest reduction in cell viability and increase in apoptosis through the reduction in the prenylation of GTPase Ras and Rho A (192)
Zoledronic acid + atorvastatin	FPPS, HMGCR	Breast cancer	Significantly impairs cancer cell adhesion on alphavbeta3 expression (193)
Zoledronic acid + paclitaxel	FPPS	Breast cancer	Has synergistic effect on tumor cell number and apoptosis (194)
Zoledronic acid + atorvastatin	FPPS, HMGCR	Breast cancer	Combined inhibition achieves a meaningful anti-tumor effect by suppressed protein geranylation (195)
Zoledronic acid + gefitinib	FPPS, EGFR	Non-small cell lung cancer	Increases the antitumor effect of gefitinib by inhibiting STAT3 expression (196)

### Targeting HMGCR for Cancer Therapy

#### Targeting HMGCR in Preclinical Cancer Therapy

Altered cholesterol metabolism is considered as a risk factor and driver of tumor growth, and is also associated with worse prognosis in a variety of cancers including breast, prostate, brain, and colorectal cancer (197, 198). Targeting HMGCR, a rate-limiting specific enzyme of cholesterol synthesis, has been identified as a potential therapeutic strategy for cancer treatment. Originally for treating cardiovascular diseases, statins like HMGCR inhibitors have become a standard of care for treating cancer patients with high cholesterol levels (199, 200) and also reduce the incidence and recurrence of various cancers, including colon (201), liver (202), and lung cancer (203). Statins can be divided mainly into two groups, depending on their origin by fungi fermentation or chemical syntheses, including type-1, mevastatin, lovastatin, simvastatin and type-2, fluvastatin, and atorvastatin (200). A number of studies have indicated that statins can inhibit cell proliferation, invasion and colony formation, and induce apoptosis to suppress tumorigenesis, tumor survival, angiogenesis and metastasis by regulating multiple signaling pathways (59, 199, 204).

In PCa xenograft mice models, simvastatin treatment at 25 μM inhibited serum-induced Akt activity, cell migration and colony formation (150). Both simvastatin and fluvastatin inhibit cell proliferation and induce apoptosis in a dose- and time-dependent manner via the downregulation of Akt/Foxo1 phosphorylation in PCa (151). Simvastatin treatment also overcomes enzalutamide-induced resistance through the inhibition of mTOR-mediated AR degradation (102).

In breast cancer, both statins and HMGCR transcriptional regulation can overcome statin resistance through the regulation of SREBP-2 cleavage (205). The findings in a 2D co-culture and a splenic mouse model demonstrate that atorvastatin suppresses breast cancer proliferation, EMT and distant metastasis (152) and also induces autophagy (153), which is related to regulating PTEN/Akt and Ras homolog family member B pathways (154). In breast cancer stem-like cells, statins at non-toxic doses significantly alter a shared cluster of 37 genes, including the Hippo, Notch, and Wnt pathways, to hold back EMT processes (155). Simvastatin induces breast cancer cell death through the deactivation of PI3K/Akt and MAPK/Erk signals (156) and also prevents triple-negative breast cancer proliferation and metastasis through Foxo3a phosphorylation (157) or HSP90 acetylation (158). Another statin, pitavastatin, can slow breast cancer-induced bone metastasis and reduce urine-derived volatile organic compounds through the mevalonate pathway (159).

Increasing evidence demonstrates the anticancer effects of statins including atorvastatin, lovastatin, and fluvastatin against lung cancer by decreasing proliferative and migratory capacity and inducing apoptosis, which is mediated by SphK1 (160), COX-2/PPARγ (161), TGF-β RII/Erk (162), and other key pathways (204). Lung cancer cells metastasize to the bone and release RANKL, IL-6, and other factors to stimulate osteoclasts, which can be inhibited by fluvastatin through autophagy induction and osteoclastogenesis (163–165).

In hepatocellular carcinoma, *in vitro* and *in vivo* studies reveal that simvastatin induces G_0_/G_1_ arrest by upregulating p21 and p27, activating AMPK and inhibiting the STAT3-Skp2 axis in HCC (166). Other studies report that TAZ, Notch1 or ROCK expression are also involved in the anti-proliferative effects of statins against HCC (167–169).

Fluvastatin has potent anti-cancer effects against renal cell carcinoma through the suppression of the Akt/mTOR signaling cascade (170) and induces lymphoma cell apoptosis by promoting ROS generation and regulating the Akt, Erk, and p38 signaling pathways via the inhibition of mevalonate metabolic products (171). Combination therapy studies demonstrated that statins combined with chemical molecules, including doxorubicin, celecoxib, venetoclax, metformin, or a MEK inhibitor, AZD6244, can synergistically suppress tumor growth in prostate, ovarian, endometrial, or pancreatic cancers, respectively (172–176). Overall, these findings suggest that statins alone or combined with other drugs inhibit the mevalonate pathway to achieve anti-cancer effects by a variety of molecular mechanisms (Table 2).

#### Targeting HMGCR in Clinical Cancer Therapy

Currently, statins are in use for preventing or treating cancer patients with prostate (206), breast (207), lung (208), liver (209), and other cancers (210, 211). The safety, efficacy and mortality benefits of statins have been assessed both alone and in combination therapy in clinical cancer patients studies (212–214).

In a 7.5 year follow-up of patients with PCa, statin use was associated with a decreased risk of death and delays in cancer progression, dependent on the increasing intensity of usage. However, statin use before diagnosis is not associated with PCa death risk (206). A meta-analysis of breast cancer patients indicates that statin can lower cancer-specific and all-cause mortality, which appears to be related to statin type (lipophilic or hydrophilic statin) and follow-up time (207). Seventeen studies in 98,445 patients indicate that statins potentially decrease cancer-specific mortality and promote the overall survival of patients with lung cancer in observational studies (215), which does not affect progression-free survival (208). In liver cancer, numerous studies have demonstrate decreased liver cancer mortality by statin treatment after adjusting for cholesterol level and body mass index, which is a novel approach for the prevention and treatment of HCC (209). In addition, post-diagnostic statin use is associated with improved survival of patients with other cancers, such as esophageal cancer (211) and ovarian cancer (210). Compared to statin alone, the combination of statins with therapeutic drugs such as thalidomide, idarubicin or tyrosine kinase inhibitor has synergistic effects for patients with refractory myeloma (216), acute myeloid leukemia (217) or NSCLC (218), respectively.

However, some contradictory studies indicate that statins have no protective effect on skin (219), colon (220), or other cancers in numerous clinical trials (221), which might be related to chemical nature, tumor stage and type, dose, use duration and patient characteristics. Therefore, well-defined patient information and clinical trial design need careful consideration in future studies of statins in cancer patients (222). Table 3 summarizes the detailed information about tumor type, the number of patients, and main findings from clinical studies of statins alone or combined with other therapeutic agents in patients with various cancers.

**Table 3:** Clinical findings for statins and N-BPs in different cancers.

**Treatments**	**Tumor type**	**No. of patients**	**Findings**
Statins before and after diagnosis	Prostate cancer	6,537	Statin use after diagnosis decreases the risk of cancer death only in men managed with androgen deprivation therapy (206)
Lipophilic statins	Breast cancer	197,048	Lipophilic statins are associated with decreased breast cancer-specific and all-cause mortality, which appears to be constrained by statin type and follow-up time (207)
Statins	Non-small cell lung cancer (Stage IV)	5,118	Statin use at the time of the diagnosis is associated with improved survival (215)
Statins	Lung cancer	98,445	Statins are potentially associated with the decreasing risk of mortality and the improvement of overall survival in observation studies, but not in randomized controlled trials (17 studies) (208)
Statins	Liver cancer	13,063	Statin use is associated with decreased liver cancer mortality by adjusting for cholesterol levels and body mass index (209)
Statins after diagnosis	Esophageal cancer	11,750	Statin use is associated with a decreased risk of cancer specific and all-cause mortality (211)
Statins after diagnosis	Ovarian cancer	5,416	Statin use is associated with improved survival in a large nation-wide cohort (210)
Lovastatin + Thaliadomide + dexamethasone	Refractory myeloma	91	The addition of lovastatin to the regimen of thalidomide and dexamethasone improves the response rate (216)
Pravastatin + idarubicin + cytarabine	Acute myeloid leukemia	46	The combination demonstrates an impressive response rate and has therapeutic benefit by targeting the cholesterol pathway (217)
Statins + EGFR-TKIs therapy	Non-small cell lung cancer	20,717	Statin use potentially enhances the therapeutic effect and decreases mortality in patients receiving EGFR-tyrosine kinase inhibitors (218)
Statins	Skin cancer	114,708	Statin use is not associated with skin cancer risk from 29 studies (219)
Statins	Colon cancer	740	Statin use is not associated with improved cancer-specific survival (220)
Statins, lipophilic	Various cancers	175,000	Statin therapy has no effect on the incidence or mortality in 27 large-scale trials (221)
Zoledronic acid	Metastatic castration-resistant prostate cancer	7,346	Zoledronic acid remains an important adjunctive treatment strategy in the care of metastatic cancer patients from 6 of Phase III randomized controlled trials (223)
Zoledronic acid	Breast cancer, multiple myeloma	7,396	Zoledronic acid prevents the development of skeletal-related events in bone metastatic patients and improve life quality, but has no effect of overall survival from 10 clinical studies (224)
Zoledronic acid + radiopharmaceuticals	Osteoblastic metastases from lung, breast, and prostate cancer	261	The addition of radiopharmaceuticals to zoledronic acid does not alter time to skeletal-related events or overall survival (225)
Zoledronic acid + docetaxel	Prostate cancer	662	The addition of docetaxel to zoledronic acid shows no evidence for improving survival in men with local advanced or metastatic cancer from 3 randomized controlled trials (226)

### Targeting FPPS for Cancer Therapy

#### Targeting FPPS in Preclinical Cancer Therapy

Amino-bisphosphonates (N-BPs), as FPPS inhibitors, represent another major class of inhibitors targeting the mevalonate pathway. Compared to original non-nitrogen containing bisphosphonates, N-BPs have an increased affinity to hydroxyapatite and interfere with FPPS in the mevalonate pathway (227), and are used for treating patients with osteoporosis (228) or osteolytic bone metastases (229). Several studies reveal anti-tumor effects of N-BPs apart from the inhibition of osteoclasts. Third-generation N-BPs, zoledronic acid (ZOL) and minodronate (YM529), are more potent inhibitors of FPPS than the first-generation bisphosphonates, and have been found to exhibit anti-tumor effects through inhibition of cell growth, induction of apoptosis, inhibition of angiogenesis, decrease in tumor cell adhesion to bone and other possible mechanisms in various cancers (230, 231).

In PCa, ZOL induces apoptosis through down-regulation of survivin (177), and inhibits RhoA-mediated amoeboid motility to impede metastatic lung colonization (178). In PCa stem cells, ZOL can facilitate the intrinsic apoptosis pathway to overcome chemoresistance (179). Moreover, ZOL exposure markedly induces autophagosome formation and inhibits protein prenylation for anti-prostate cancer activity (180, 181). In breast cancer, ZOL can significantly reduce the expression of cancer cell factors such as CCL2 and IDO to suppress regulatory T-cell function (182). Especially, a formed ZOL-nanoparticle restores doxorubicin cytotoxic efficacy against chemo-immunoresistant tumors by reducing metabolic flux and also lowers Ras/Erk1/2/HIF-1α axis activity to maintain cell death and immunosuppression (183). In lung cancer, *in vitro* and *in vivo* experiments demonstrate that ZOL-treated cells typically arrest the at S/G_2_/M phase with increases of cyclins and cyclin-related regulatory proteins such as Ras (184). ZOL can also inhibit Ras and Rap1A prenylation to target lung cancer (185). Similar findings in HCC demonstrate that ZOL inhibits the translocation of Ras and RhoA to reduce cell growth and prevent progression to bone metastatic lesions (186). Additionally, ZOL treatment reverts M2 macrophages to M1 phenotype for producing IFN-γ (188) or activating Vγ9Vδ2 T cells (187) to suppress tumorigenesis through the immune modulation.

Another N-BP, YM529, also exerts anti-tumor effects against various types of cancer cells, including PCa, NSCLC, and bladder cancer, by various mechanisms such as CXCR-4-induced invasion (189), down-regulation of Erk1/2 phosphorylation (190), and inhibition of Rap1A prenylation (191). In addition, N-BPs have been used in combination with chemotherapy, statins or enzyme inhibitors to achieve additive or synergistic effects by diverse mechanisms, including a reduction in protein prenylation, impairment of geranylgeranylation or inhibition of STAT3 in prostate (192), breast (193–195), and lung (196) cancers. The effects of FPPS inhibitors such as ZOL and YM529 alone and in combination with other drugs targeting multiple signaling pathways in cancer cell and xenograft models are summarized in Table 2.

#### Targeting FPPS in Clinical Cancer Therapy

Based on their strong inhibitory effect on osteoclasts, N-BPs are used to treat osteolytic bone metastases, which are frequent in advanced cancer, especially prostate and breast cancer. In PCa, ZOL has become an established first-line or adjunctive treatment in bone-targeted therapy for metastatic castration-resistant progression (223, 232). Though ZOL delays skeletal-related events (SREs), it reportedly has no effect on overall survival, other disease-oriented parameters, or radiographic progression improvement. It remains an important adjunctive treatment strategy in the care of metastatic castrate-resistant PCa patients (223). Findings in clinical trials indicate that the beneficial effect of ZOL on bone metastasis from advanced prostate cancer might be related to long-term therapy, generally for more than 2 years (226). Similarly, ZOL can prevent the development of SREs in bone metastatic patients with breast cancer and improve quality of life, although with no effect on overall survival (224, 225). However, long-term side effects of ZOL, such as impaired renal function and bone pain need to be taken into consideration for treatment decisions (233). Findings in clinical studies of ZOL treatment alone or in combination are summarized in Table 3.

Overall, the SREBP-2-regulated mevalonate pathway is a crucial regulator for tumor progression and a promising therapeutic target. Targeting SREBP-2, HMGCR or FPPS has become an attractive strategy for cancer therapy. Preclinical (Table 2) and clinical (Table 3) studies demonstrate that fatostatin, statins, ZOL, and YM529, alone or in combination with chemotherapy or other drugs, have anti-tumor effects through a variety of molecular mechanisms.

## Conclusions

This review has summarized the structure, activation and regulation of SREBP-2 by multiple signaling pathways. SREBP-2 and its regulated enzymes from the mevalonate pathway, including HMGCR, FPPS, SQS, and DHCR4, participate in the progression of various cancers including prostate, breast, lung, and hepatocellular cancer, and thus are important potential therapeutic targets. Importantly, preclinical and clinical research has demonstrated that fatostatin, statins, and N-BPs targeting SREBP-2, HMGCR, and FPPS, respectively, alone or in combination with other drugs, are used for the treatment of different cancers. This review provides new insights into the critical role of the SREBP-2-regulated mevalonate pathway in cancer and its potential for targeted cancer therapy.

As a metabolic reprogramming process, the SREBP-2-regulated mevalonate pathway has a high-degree of similarity with glucose or glutamine metabolism and links them together to participate in cancer progression. Based on the function of SREBP-2 in cholesterol biosynthesis, it is necessary to develop new strategies specifically targeting SREBP-2 to treat various cancers with dysfunctional cholesterol metabolism. Combination treatments simultaneously targeting SREBP-2 and its regulated enzymes from the mevalonate pathway may achieve beneficial effects for cancer treatment and prevention, and represent important future directions in ongoing research.

## Author Contributions

LX, XL, and BW: conceptualization. LX: writing—original draft preparation. HQ, HZ, LD, and QH: writing—review and editing of different sections. DZ, BW, and XL: supervision. All authors have read and agreed to the published version of the manuscript.

## Conflict of Interest

The authors declare that the research was conducted in the absence of any commercial or financial relationships that could be construed as a potential conflict of interest.
